# Functional visualization and disruption of targeted genes using CRISPR/Cas9-mediated eGFP reporter integration in zebrafish

**DOI:** 10.1038/srep34991

**Published:** 2016-10-11

**Authors:** Satoshi Ota, Kiyohito Taimatsu, Kanoko Yanagi, Tomohiro Namiki, Rie Ohga, Shin-ichi Higashijima, Atsuo Kawahara

**Affiliations:** 1 Laboratory for Developmental Biology, Center for Medical Education and Sciences, Graduate School of Medical Science, University of Yamanashi, Shimokato 1110, Chuo, Yamanashi, 409-3898, Japan; 2 National Institutes of Natural Sciences, Okazaki Institute for Integrative Bioscience, National Institute for Basic Biology, Okazaki, Aichi, 444-8787, Japan

## Abstract

The CRISPR/Cas9 complex, which is composed of a guide RNA (gRNA) and the Cas9 nuclease, is useful for carrying out genome modifications in various organisms. Recently, the CRISPR/Cas9-mediated locus-specific integration of a reporter, which contains the Mbait sequence targeted using *Mbait*-gRNA, the hsp70 promoter and the eGFP gene, has allowed the visualization of the target gene expression. However, it has not been ascertained whether the reporter integrations at both targeted alleles cause loss-of-function phenotypes in zebrafish. In this study, we have inserted the Mbait-hs-eGFP reporter into the *pax2a* gene because the disruption of *pax2a* causes the loss of the midbrain-hindbrain boundary (MHB) in zebrafish. In the heterozygous Tg[pax2a-hs:eGFP] embryos, MHB formed normally and the eGFP expression recapitulated the endogenous *pax2a* expression, including the MHB. We observed the loss of the MHB in homozygous Tg[pax2a-hs:eGFP] embryos. Furthermore, we succeeded in integrating the Mbait-hs-eGFP reporter into an uncharacterized gene *epdr1*. The eGFP expression in heterozygous Tg[epdr1-hs:eGFP] embryos overlapped the *epdr1* expression, whereas the distribution of eGFP-positive cells was disorganized in the MHB of homozygous Tg[epdr1-hs:eGFP] embryos. We propose that the locus-specific integration of the Mbait-hs-eGFP reporter is a powerful method to investigate both gene expression profiles and loss-of-function phenotypes.

The type II clustered regularly interspaced short palindromic repeats (CRISPR)/CRISPR associated protein (Cas) system has become a powerful genetic tool to manipulate various genome modifications in target sites^1^,^2^,^3^. In this system, the targeted locus is recognized and cleaved by the complex of three components, CRISPR RNA (crRNA), *trans*-activating crRNA (tracrRNA) and Cas9 nuclease^1^. The crRNA contains complementary RNA sequences that interact with the targeted genome sequences followed by the protospacer-adjacent motif (PAM) sequence NGG (N: any nucleotide), and the tracrRNA guides crRNA to the Cas9 nuclease. A single guide RNA (gRNA) combining the crRNA and tracrRNA is widely used as a simple two-component (gRNA/Cas9) system^2^,^3^. DNA double-strand breaks (DSBs) induced by CRISPR/Cas9 are repaired using DNA repair mechanisms, including non-homologous end joining (NHEJ), microhomology-mediated end joining (MMEJ) and homologous recombination (HR)^4^,^5^. NHEJ is an error-prone mechanism, and it often results in insertion and/or deletion (indel) mutations at the target site, leading to frameshift-mediated gene disruption. MMEJ can connect the exposed ends of microhomology sequences (3–25 bases) in the targeted locus^6^,^7^. In the presence of a donor DNA template containing long homologous sequences around the target site, DSBs can be repaired by the HR-mediated integration of the homologous fragment derived from the donor vector^8^,^9^,^10^.

Recently, Auer *et al*. have reported the CRISPR/Cas9-mediated integration of reporter genes at targeted loci in zebrafish^11^. The authors designed a donor vector containing a bait sequence targeted by gRNA/Cas9 and observed the efficient genomic integration of the donor vector when concurrent cleavages of the donor vector and the targeted genomic locus were produced by gRNAs/Cas9. In fact, the authors succeeded in converting eGFP transgenic lines to Gal4 transgenic lines using this homology-independent knock-in system. More recently, Kimura *et al*. have modified the system and achieved efficient genomic integrations of reporter/driver vectors in zebrafish^12^. The authors used the modified donor vector containing the Mbait sequence derived from the rat *Mcr4* gene, the hsp70 promoter and the reporter/driver genes, and they demonstrated the CRISPR/Cas9-mediated locus-specific integration of the reporter, resulting in the continuous visualization of target gene expression. However, it remains uncertain whether the reporter gene integrations in both targeted alleles lead to loss-of-function phenotypes in zebrafish. In this study, we demonstrated that the locus-specific integration of the Mbait-hs-eGFP reporter by CRISPR/Cas9 is a powerful genetic method not only for visualization of continuous target gene expression but also for conducting a loss-of-function analysis of gene of interest in zebrafish.

## Results

### Genomic integration of the Mbait-hs-eGFP reporter into the *pax2a* locus

**Figure 1:**
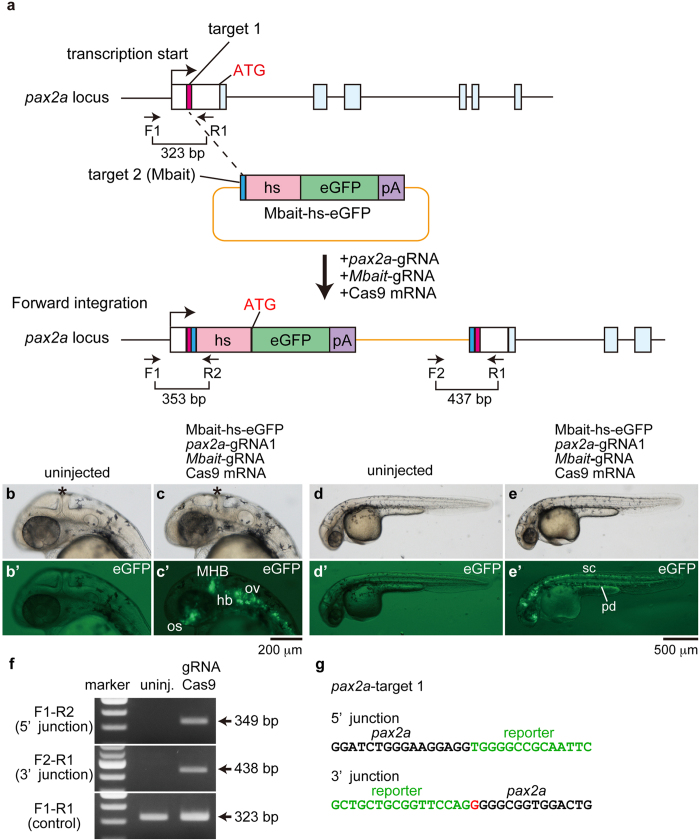
Targeted genomic integration of the Mbait-hs-eGFP reporter into the *pax2a* locus using the CRISPR/Cas9. (**a**) A schematic representation of the *pax2a* locus containing the 5′ untranslated region (white box) and the *pax2a*-gRNA target sites (pink box), and the reporter construct consisting of the *Mbait*-gRNA target site (blue box), the hsp70 promoter (hs, light red box), the eGFP gene (green box) and the polyA signal (pA, purple box). The Mbait-hs-eGFP reporter was co-injected with *pax2a*-gRNA1, *Mbait*-gRNA and Cas9 mRNA into 1-cell stage zebrafish embryos. Concurrent cleavage of the targeted genomic locus and the Mbait-hs-eGFP reporter resulted in the integration of the reporter by non-homologous end joining. The scheme shows forward integration of the reporter. (**b**,**b**’,**d**,**d**’) An uninjected control embryo around 32 hpf shows no eGFP expression. (**c**,**c**’,**e**,**e**’) The expression of eGFP around 32 hpf was detected in the midbrain-hindbrain boundary (MHB)(asterisk), the optic stalk (os), the otic vesicles (ov), the hindbrain neurons (hb), the spinal cord (sc) neurons and the pronephric duct (pd) in the injected embryo at 1 dpf. (**f**) The integration of the reporter into the *pax2a* gene was determined by genomic PCR using the *pax2a*-specific and reporter-specific primers. The targeted positions of the primers are shown in (**a**). (**g**) The sequences of the 5′ junction and the 3′ junction at the integration site of the eGFP-positive embryo (**c**,**c**’). A 4-bp deletion and a 1-bp insertion were observed at the 5′ junction and the 3′ junction, respectively. The red letter represents the inserted nucleotide. (**b**–**e**,**b**’–**e**’) Lateral view with anterior to the left and dorsal to the top. Black letters and green letters represent *pax2a* sequences and the reporter sequences, respectively.

To examine whether the CRISPR/Cas9-mediated reporter integration into both alleles of the target gene causes a loss-of-function phenotype, we inserted a reporter into the *paired box 2a* (*pax2a*) gene because the disruption of *pax2a* results in the loss of the midbrain-hindbrain boundary (MHB). The mutant is well known as the *no isthmus* (*noi*) mutant^13^,^14^. The Mbait-hs-eGFP reporter contains the Mbait sequence, the hsp70 promoter (hs), and the eGFP gene (Fig. 1a). Because the Mbait sequence derived from the rat *Mcr4* gene shows no homology to the zebrafish genome sequence, the complex of *Mbait*-gRNA and Cas9 selectively cleaves the Mbait-hs-eGFP reporter^12^. We prepared three gRNAs targeting the 5′ untranslated region (UTR) of *pax2a*. When the Mbait-hs-eGFP reporter was co-injected with *pax2a*-gRNA1, *Mbait*-gRNA and Cas9 mRNA into 1-cell stage zebrafish embryos, we observed eGFP expression overlapping the *pax2a* expression domains in 1.2% of the injected embryos (Fig. 1, Supplementary Table S1). Thus, eGFP expression was detected in the MHB, the optic stalk, the otic vesicles, the pronephric duct, and the hindbrain and spinal cord neurons where endogenous *pax2a* is expressed^15^. Subsequently, we prepared genomic DNA from the eGFP-positive embryos and performed genomic PCR using *pax2a*-specific and vector-specific primers. We detected the locus-specific integration in the *pax2a* gene (Fig. 1f). A sequence analysis revealed that the reporter was integrated into the 5′UTR of the *pax2a* gene (Fig. 1g). We found that the indel mutation frequency of *pax2a*-gRNA1/Cas9 mRNA-injected embryos was 40% (Supplementary Fig. S1). In the eGFP-positive F0 embryos (Fig. 1), we failed to detect the loss of the MHB that occurs in the *noi* mutant, suggesting that the efficacy of the reporter knock-in was not sufficient to induce the *noi* phenotype. We examined the genome-editing activities of *pax2a*-gRNA2 and *pax2a*-gRNA3. The somatic mutation frequencies of *pax2a*-gRNA2/Cas9-injected and *pax2a*-gRNA3/Cas9-injected embryos were 6.7% and 7.7%, respectively (Supplementary Fig. S1). When *pax2a*-gRNA2 or *pax2a*-gRNA3 was co-injected with the Mbait-hs-eGFP reporter, *Mbait*-gRNA and Cas9 mRNA, the eGFP expression was detected in the MHB, the optic stalk and the hindbrain neurons. The frequencies of eGFP-positive embryos recapitulating *pax2a* expression were 2.2% in the *pax2a*-gRNA2/Cas9-injected embryos and 1.4% in the *pax2a*-gRNA3/Cas9-injected embryos (Supplementary Fig. S2 and Table S1). It is not clear why the frequencies of the reporter integration judged by eGFP expression were similar in the condition that the frequencies of indel mutations for the three *pax2a*-gRNAs judged by genomic sequencing were different. We found possible off-target sites for *pax2a*-gRNA1 and *pax2a*-gRNA2 in the cording regions of candidate genes and examined genome modifications by the heteroduplex mobility assay (HMA). As the result, the off-target effects for the candidate sites were marginal (Supplemental Fig. S3). Genomic PCR revealed the genomic integration of the reporter into the *pax2a* locus, and a sequence analysis verified the integration-containing indel mutations at the 5′ and 3′ junctions of the *pax2a* gene (Supplementary Fig. S2). We failed to observe the *noi* phenotype in the *pax2a*-gRNA2/Cas9 and *pax2a*-gRNA3/Cas9-injected embryos. Thus, we succeeded in the integration of the Mbait-hs-eGFP reporter into the *pax2a* gene, however, the knock-in efficacy in the injected F0 embryos was not sufficient to induce the *noi* mutant phenotype.

### Generation and characterization of Tg[pax2a-hs:eGFP]

**Figure 2:**
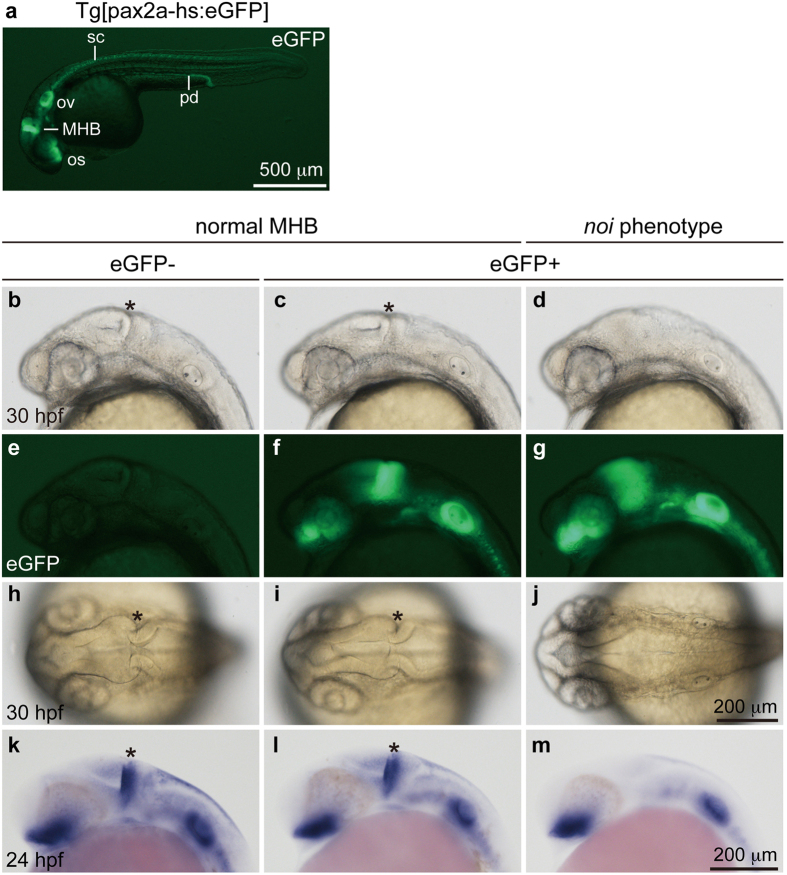
eGFP expression and *noi* phenotype in Tg[pax2a-hs:eGFP] embryos. (**a**) A heterozygous Tg[pax2a-hs:eGFP] embryo at 30 hpf. The expression of eGFP was detected in the MHB, the optic stalk (os), the otic vesicles (ov), the spinal cord (sc) neurons and the pronephric duct (pd). (**b**–**j**) The 30 hpf F2 embryos derived from mating of heterozygous Tg[pax2a-hs:eGFP] F1 fish. (**b**,**e**,**h**) A wild-type embryo. The isthmus is formed at the MHB (asterisk). (**c**,**f**,**i**) A heterozygous Tg[pax2a-hs:eGFP] embryo. The isthmus is formed normally. (**d**,**g**,**j**) A homozygous Tg[pax2a-hs:eGFP] embryo. eGFP expression around the MHB was anteriorly expanded. The isthmus was not formed in the homozygous Tg[pax2a-hs:eGFP] embryo. Genotyping was performed using genomic PCR. (**k**–**m**) Whole-mount *in situ* hybridization using anti-sense *pax2a* RNA probe at 24 hpf. The *pax2a* expression in the MHB was detected in the wild-type (**k**) and the heterozygous (**l**) embryos, but not in the homozygous embryos (**m**). (**a–g**,**k–m)** Lateral view with anterior to the left and dorsal to the top. (**h**–**j**) Dorsal view with anterior to the left.

**Figure 3:**
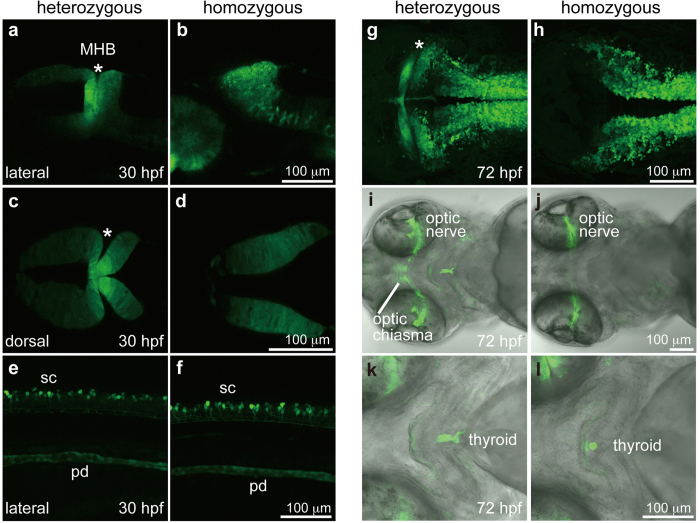
eGFP expression visualized by the endogenous *pax2a* enhancer activity. (**a**,**c**) The MHB (asterisk) of a heterozygous Tg[pax2a-hs:eGFP] embryo at 30 hpf. (**b**,**d**) The midbrain and hindbrain of a homozygous Tg[pax2a-hs:eGFP] embryo at 30 hpf. (**e**,**f**) The spinal cord neurons (sc) and pronephric duct (pd) of heterozygous (**e**) and homozygous (**f**) Tg[pax2a-hs:eGFP] embryos at 30 hpf. (**g**) The MHB of the heterozygous Tg[pax2a-hs:eGFP] embryo at 72 hpf. (**h**) The midbrain and hindbrain of the homozygous Tg[pax2a-hs:eGFP] embryo at 72 hpf. (**i**,**j**) The optic nerves of heterozygous (**i**) and homozygous (**j**) Tg[pax2a-hs:eGFP] embryos at 72 hpf. (**k**,**l**) The thyroids of heterozygous (**k**) and homozygous (**l**) Tg[pax2a-hs:eGFP] embryos at 72 hpf. All data were obtained by confocal microscopy. (**a**,**b**,**e**,**f**) Genotyping was performed using genomic PCR. Lateral view with anterior to the left and dorsal to the top. (**c**,**d**,**g**,**h**) Dorsal view with anterior to the left. (**i–l**) Ventral view with anterior to the left.

To establish a reporter knock-in transgenic line, we injected *pax2a*-gRNAs (*pax2a*-gRNA1–3; 10 pg each, total 30 pg), *Mbait*-gRNA, Mbait-hs-eGFP and Cas9 mRNA into 1-cell stage embryos. We found that 5% of the injected embryos exhibited eGFP expression overlapping endogenous *pax2a* expression (Supplementary Table S1), and we then raised the embryos to adulthood. When potential F0 founders were mated with wild-type, one of 20 founders produced eGFP-positive embryos with a germline transmission frequency of 32% (Supplementary Table S2). We observed that eGFP expression of heterozygous Tg[pax2a-hs:eGFP] embryos completely recapitulated the endogenous *pax2a* expression domains, including the MHB, the optic stalk, the otic vesicles, the pronephric duct, and the hindbrain and spinal cord neurons (Fig. 2a). Furthermore, confocal imaging revealed eGFP expression in the interneurons of the spinal cord, the optic nerve and the thyroid (Fig. 3). A sequence analysis verified the genomic integration into the *pax2a* gene, indicating the establishment of a Tg[pax2a-hs:eGFP] line (Supplementary Fig. S4).

Next, we examined the homozygous phenotype by mating heterozygous male and female Tg[pax2a-hs:eGFP] fish. We found that 21% (32/153) of the F2 embryos exhibited the loss of the MHB (Fig. 2), a phenotype identical to that of the *noi* mutant^13^,^14^, suggesting that the locus-specific integration of the reporter in both alleles causes the loss-of-function phenotype. Although the *pax2a* expression in the MHB of the *noi* mutant is lost at the mid-somitogenesis stages^13^, eGFP expression in the homozygous Tg[pax2a-hs:eGFP] embryo at 30 hours post-fertilization (hpf) was still detected in the midbrain (Fig. 2g). Importantly, the *pax2a* expression in the MHB was abolished in the homozygous embryo (Fig. 2k–m), while the *pax2a* expression in the optic stalk and hindbrain was detected: these phenotypes were identical to the *noi*/*pax2.1* mutant phenotypes^13^. Reverse transcription (RT)-PCR revealed that the *pax2a* transcripts containing exon 1 and exon 2 were decreased in the homozygous embryos, while the *pax2a* transcripts containing exon 3 and exon 4 were comparably detected (Supplementary Fig. S5), suggesting the suppression of functional *pax2a* transcripts in the homozygous embryos. We classified the F2 embryos into three groups based on the eGFP expression and the MHB abnormality, and we performed genomic PCR to determine the genotypes of the embryos. We confirmed that all embryos analyzed that exhibited the *noi* mutant phenotype with strong eGFP expression were homozygous, showing that the loss of the MHB was caused by the integration of the reporter at both alleles of the *pax2a* locus (Supplementary Fig. S6).

The reporter integration in the Tg[pax2a-hs:eGFP] embryos enabled us to investigate the continuous morphological defects induced by the loss of *pax2a* function in early embryogenesis. A confocal imaging analysis revealed that the eGFP-positive cells in homozygous embryo at 30 hpf expanded anteriorly, while eGFP expression of the heterozygous embryo was restricted to the MHB (Figs 2 and 3a–d). The disorganization of the MHB was also observed in the homozygous embryos at 72 hpf (Fig. 3g,h). Importantly, an abnormal projection of the optic axons in the homozygous embryo, but not in the heterozygous embryo, was continuously visualized by eGFP expression, showing abnormal optic nerve formation in the optic chiasma (Fig. 3i,j). There was no obvious difference between heterozygous and homozygous embryos in the eGFP-positive cells of the spinal cord interneurons and of the pronephric duct (Fig. 3e,f). Interestingly, we found that homozygous embryo exhibited abnormal morphology in the posterior part of the thyroid, presumably the thyroid follicles (Fig. 3k,l), as previously reported^16^. We emphasize that the results of the morphological defects are continuously visualized by eGFP expression in the reporter-integrated homozygous embryo.

### Generation and characterization of Tg[epdr1-hs:eGFP]

**Figure 4:**
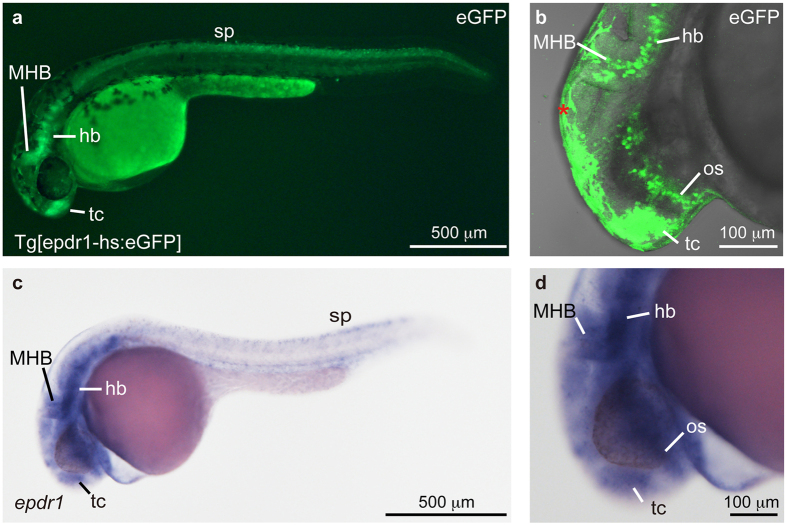
Establishment of Tg[epdr1-hs:eGFP]. (**a**,**b**) A heterozygous Tg[epdr1-hs:eGFP] embryo. eGFP expression was detected in the telencephalon (tc), the midbrain-hindbrain boundary (MHB), the hindbrain (hb) and spinal neurons (sp) at 30 hpf. The *epdr1* expression in the optic stalk (os) was detected by confocal microscope (**b**). Autofluorescence of epidermis was detected (*). (**c**,**d**) Expression of *epdr1* in the tc, the os, the MHB, the hb and the sp at 24 hpf. The expression of *epdr1* was examined by whole-mount *in situ* hybridization using an anti-sense *epdr1* RNA probe.

Recently, we developed the ready-to-use CRISPR/Cas9 system composed of synthetic crRNA and tracrRNA together with recombinant Cas9 protein^17^. Using this system, we visualized the expression pattern of an uncharacterized gene, *ependymin related 1* (*epdr1*), in F0 embryos^17^. Genome editing activities were determined by genomic PCR and genomic sequencing (Supplementary Figs 7 and 8). In this study, we established and characterized Tg[epdr1-hs:eGFP] zebrafish. When we mated potential F0 founders with wild-type, we found that one out of the 58 F0 founders produced eGFP-positive F1 embryos with a germline transmission frequency 46% (Supplementary Table S2). In the heterozygous Tg[epdr1-hs:eGFP] embryos, eGFP expression was detected in the telencephalon, the optic stalk, the MHB, and the hindbrain and spinal neurons (Fig. 4a,b). The eGFP expression domains recapitulated the endogenous *epdr1* expression domains (Fig. 4c,d), and the reporter responded to the enhancer activity of the *epdr1* gene. To perform PCR genotyping, we prepared genomic DNA from the eGFP-positive embryos, and confirmed that the Mbait-hs-eGFP reporter was integrated into the *epdr1* locus (Supplementary Fig. 9). RT-PCR analysis revealed that the *epdr1* transcripts containing exon 1 and exon 2 were suppressed in the homozygous embryos (Supplementary Fig. 10).

**Figure 5:**
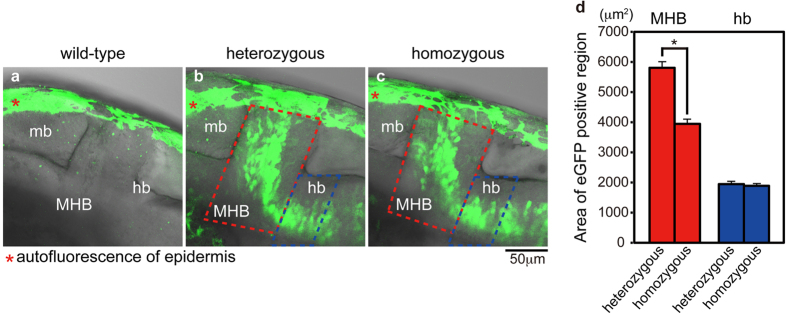
Functional analysis of Tg[epdr1-hs:eGFP]. (**a**) Autofluorescence of epidermis in wild-type embryo (red asterisk). (**b**,**c**) The distribution of eGFP-positive cells in the midbrain (mb) and the hindbrain (hb) of heterozygous (**b**) and homozygous (**c**) Tg[epdr1-hs:eGFP] embryos at 30 hpf (lateral view, anterior left) (confocal images). Autofluorescence in epidermis is indicated by red asterisk. (**d**) The area of eGFP-positive cells of heterozygous and homozygous Tg[epdr1-hs:eGFP] embryos in the MHB (red dashed line) and anterior part of hindbrain (blue dashed line) in the visual field (*N* = 14 each). Error bars indicate standard error of the mean (SEM). Statistical significance was determined using Student’s t-test. *P < 0.05.

**Figure 6:**
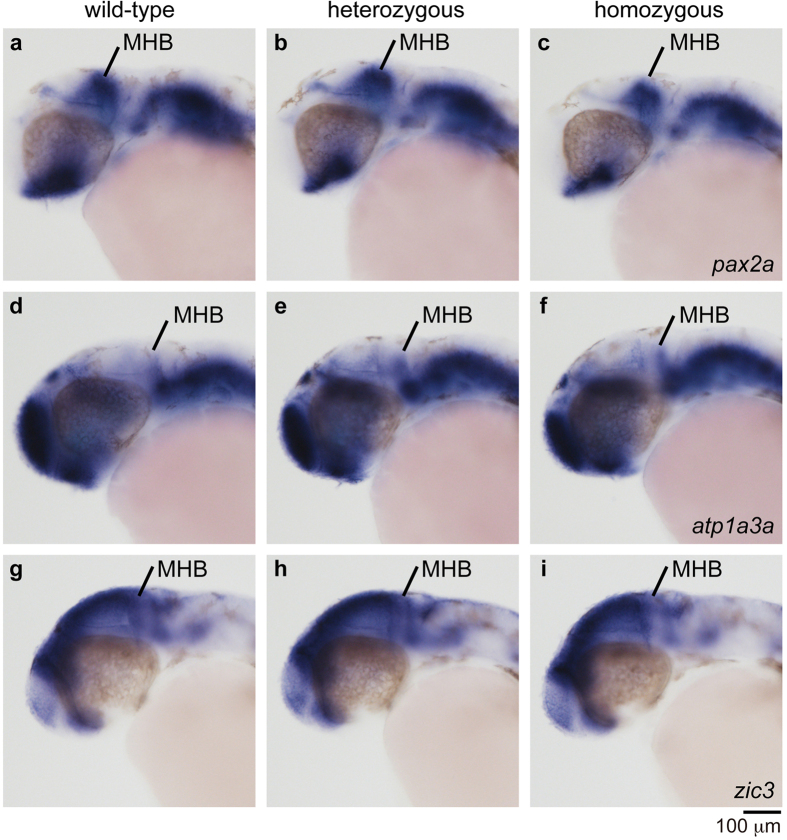
Expression of neural genes in the midbrain and the hindbrain of Tg[epdr1-hs:eGFP] embryos. The expression of neural marker genes was examined by whole-mount *in situ* hybridization using the indicated anti-sense RNA probes at 30 hpf. The expression of *pax2a* in the MHB and the optic stalk of the homozygous Tg[epdr1-hs:eGFP] embryo was slightly decreased compared with that of the wild-type or the heterozygous Tg[epdr1-hs:eGFP] embryos. The expression of *atp1a3a* and *zic3* was not affected.

Next, we examined the morphological difference between heterozygous and homozygous Tg[epdr1-hs:eGFP] embryos at 30 hpf. We found that the distribution of eGFP-positive cells was disorganized in the MHB of homozygous embryos (Fig. 5). Furthermore, we investigated the expression patterns of neural genes by whole-mount *in situ* hybridization at 30 hpf. We found that the *pax2a* expression in the MHB and the optic stalk was slightly decreased in the homozygous Tg[epdr1-hs:eGFP] embryos, whereas the *atp1a3a* and *zic3* expression did not differ between heterozygous and homozygous Tg[epdr1-hs:eGFP] embryos (Fig. 6). RT-PCR analysis revealed the *pax2a* transcripts were weakly decreased in the homozygous Tg[epdr1-hs:eGFP] embryos (Supplementary Fig. 11). Thus, the targeted genomic integration of the reporter would be useful for analysing the expression profiles and loss-of-function phenotypes of uncharacterized genes.

## Discussion

In this study, we demonstrated the functional visualization and disruption of targeted genes by the CRISPR/Cas9-mediated eGFP reporter integration in zebrafish. This method has several advantages compared with conventional knock-out methods using genome-editing technologies^18^,^19^. First, screening of reporter knock-in lines with our method is easy compared with the identification of knock-out alleles by conventional methods. We only need to search the embryos expressing eGFP in the domains where the endogenous target gene is expressed, whereas evaluating indel mutations from conventional knock-out methods requires genomic PCR and sequencing. Furthermore, we can easily identify homozygous embryos because eGFP expression in homozygous embryos is more intense than in heterozygous embryos (Supplementary Fig. S9). Second, the development of morphological defects in the reporter-integrated homozygous embryos can be continuously visualized by eGFP expression. As previously reported^16^,^20^, abnormal axon projection in the forebrain and abnormal formation of the thyroid follicles in the *pax2a*/*noi* mutants have been examined by immunohistochemistry of the fixed embryos. By contrast, the morphological abnormalities in the reporter-integrated homozygous live embryos were visualized and analysed as eGFP expression. Third, we can isolate and analyse eGFP-positive cells from defective organs of the reporter-integrated homozygous embryos using a cell sorter, which is useful for analysing loss-of-function of the target gene.

We succeeded in establishing heterozygous Tg[pax2a-hs:eGFP] individuals who expressed eGFP in the MHB, the optic stalk, the otic vesicle, the pronephric duct, and the hindbrain and spinal cord neurons (Figs 2 and 3). Those eGFP expression domains recapitulated the endogenous *pax2a* expression domains. Although it is possible that the endogenous *pax2a* basal promoter regulated the eGFP expression, we assumed that the hsp70 promoter precisely received the enhancer and/or suppressor activity in the *pax2a* gene. Picker *et al*. previously reported pGFP5.3 transgenic zebrafish generated by the insertion of the identified *pax2a* promoter/enhancer-eGFP into a certain locus of the zebrafish genome^21^. This transgenic line expressed eGFP in the MHB, the otic vesicle, the hindbrain and spinal cord interneurons, but not in the pronephric duct. In addition, ectopic eGFP expression was detected in the telencephalon, the diencephalon, and in rhombomere 3 and 5, indicating that this pGFP5.3 line partly mimicked endogenous *pax2a* expression. An important point is that the pGFP5.3 line is not acceptable for a loss-of-function analysis because the *pax2a* gene is intact in the line. By contrast, eGFP expression in the heterozygous Tg[pax2a-hs:eGFP] completely overlapped the endogenous *pax2a* expression domains. We found that the homozygous Tg[pax2a-hs:eGFP] embryos exhibited the loss of the MHB, an abnormal projection of the optic axons and disorganization of the thyroid; these phenotypes are identical to the those of *pax2a*/*noi* mutant^13^,^14^,^16^,^20^. Furthermore, we can analyse the development of morphological abnormalities by eGFP expression in live homozygous embryos. Therefore, the reporter-integrated heterozygous embryo is useful for real-time visualization of target gene expression, and the loss-of-function phenotype of the target gene can be continuously monitored by eGFP expression in the reporter-integrated homozygous embryo. Consistent with our findings, Hoshijima *et al*. have recently reported that promoter-less reporter construct integrations by TALEN-mediated homologous recombination cause loss-of-function phenotypes for the target genes^22^.

Our method is also acceptable for a functional analysis of an uncharacterized gene. We established heterozygous Tg[epdr1-hs:eGFP] embryos and found that eGFP was expressed in the telencephalon, the optic stalk, the MHB, and the hindbrain and spinal neurons, and those eGFP expression domains recapitulated the endogenous *epdr1* expression domains (Fig. 4). In the homozygous Tg[epdr1-hs:eGFP] embryos, the distribution of eGFP-positive cells in the MHB, but not the hindbrain, was disorganized (Fig. 5). Furthermore, the *pax2a* expression, but not the *atp1a3a* or *zic3* expression, was slightly decreased in homozygous Tg[epdr1-hs:eGFP] embryo (Fig. 6 and Supplementary Fig. 11). As shown in Supplementary Movies 1,2,3, the touch response in homozygous Tg[epdr1-hs:eGFP] embryo was normally observed at 2 dpf. The function of *epdr1 in vivo* is still unclear; therefore, further analyses will be required to ascertain whether the disruption of *epdr1* affects neural development and function. In conclusion, we propose that locus-specific reporter integration by CRISPR/Cas9 is a powerful genetic tool not only for continuous visualization of target gene expression but also for conducting a loss-of-function analysis of target genes in zebrafish.

## Methods

### Ethics Statement

The methods in this study were performed in accordance with the fundamental guidelines for proper conduct of animal experiment and related activities of the Ministry of Education, Culture, Sports, Science and Technology in Japan. All experimental protocols in this study were approved by the institutional animal care and use committee of Yamanashi University (approval identification number: A25–28).

### Preparation of gRNAs and Cas9 mRNA

The targeted genomic sequences and oligonucleotides used in this study are listed in Supplementary Table S3. To construct the pDR274-*pax2a*-gRNA1–3, sense and anti-sense oligonucleotides were annealed and inserted into the BsaI-cleaved pDR274 vector^19^. The Cas9 plasmid pCS2-hSpCas9 was kindly provided by Dr. Kinoshita (Kyoto University)^23^. The gRNA was transcribed from the linearized pDR274-derived vector using MAXIscript T7 Kit (Thermo Fisher Scientific). The Cas9 mRNA was transcribed from the linearized pCS2-hSpCas9 using mMESSAGE mMACHINE SP6 Kits (Thermo Fisher Scientific).

### Synthetic crRNA and tracrRNA, and recombinant Cas9 protein

For the targeted insertion of the Mbait-hs-eGFP reporter in the *epdr1* locus, we used the ready-to-use CRISPR/Cas9 system composed of synthetic crRNA and tracrRNA, and recombinant Cas9 protein^17^. Both crRNAs and tracrRNA were obtained from FASMAC, whereas recombinant Cas9 protein was obtained from Toolgen.

### Microinjection

For the targeted insertion of the Mbait-hs-eGFP reporter into the *pax2a* locus, Mbait-hs-eGFP (25–50 pg), *pax2a*-gRNA1–3 (10 pg each, total 30 pg), *Mbait*-gRNA (10 pg) and Cas9 mRNA (100 pg) were co-injected into 1-cell stage zebrafish embryos. The injection condition of the Mbait-hs-eGFP reporter at the *epdr1* locus was described previously^17^.

### Genomic PCR, genomic sequencing and heteroduplex mobility assay (HMA)

To prepare the genomic DNA, uninjected or eGFP-positive embryos at 1-day post-fertilization (1 dpf) or 2 dpf were incubated in 108 μl of 50 mM NaOH at 98 °C for 10 min. Then, 12 μl of 1 M Tris-HCl (pH 8.0) was added to the solution^17^. Genomic fragments for the targeted genomic locus were amplified from the solution (1 μl) using PCR (Supplementary Table S4). The resultant PCR fragments were sub-cloned into the pGEM-T Easy vector (Promega) and genomic sequences were determined by sequence analysis. For HMA analysis, DNA fragments (100–150 bp) containing the targeted genomic locus were amplified by PCR using the locus-specific primers (Supplementary Table S4)^24^. The resulting PCR amplicons were electrophoresed on a 12.5% polyacrylamide gel.

### Confocal imaging analysis

For confocal imaging, Tg[pax2a-hs:eGFP] or Tg[epdr1-hs:eGFP] embryos were anesthetized and mounted in 1.2% low melting point agarose. Confocal images were collected using the Olympus FV1200 confocal microscope (20× water immersion objective) at 1 μm intervals with a 488 nm laser to create a stack in the *z*-axis. To measure eGFP-positive area in the MHB and hindbrain of heterozygous or homozygous Tg[epdr1-hs:eGFP] embryos, confocal images were generated by integrating sequential sections along the *z* direction. The eGFP-positive areas in the MHB (surrounded by red dotted lines) and the anterior part of hindbrain (surrounded by blue dotted lines) of the visual field were measured using photoshop (Adobe).

### Whole-mount *in situ* hybridization and reverse transcription (RT)-PCR

We examined the expression of *epdr1*^17^, *pax2a*^13^, *atp1a3a* and *zic3*^25^. The *pax2a* plasmid contains the full-length of *pax2a* cDNA. The *atp1a3a* gene was isolated from the cDNA of 1 dpf zebrafish using the atp1a3a-F and atp1a3a-R primers (Supplementary Table S4). The resultant PCR products were digested by BamHI and XbaI, and inserted into an BamHI-XbaI-cleaved pCS2P+ vector. Anti-sense RNA probes labelled with digoxigenin were synthesized using the RNA labelling kit (Roche). Whole-mount *in situ* hybridization was performed as previously described^26^. For RT-PCR analysis, total RNAs (10 embryos) were prepared by TRIzol (Thermo Fisher Scientific) at 30 hpf stage embryos. Total RNAs were treated with reverse transcriptase (superscript III)(Thermo Fisher Scientific), and the transcripts for target genes were amplified by PCR using individual gene-specific primers (Supplementary Table S4). The resulting PCR amplicons were electrophoresed on agarose gels or polyacrylamide gels. The amount of PCR products was determined by optical density measurement.

### Behavioural analysis

Embryos at 2 dpf were mounted in 1.2% low melting point agarose. The preparations were submerged in the E3 medium and the head and tail of the embryos were freed from the agarose. Tactile stimuli were delivered to the head by using a needle. Embryonic behaviours were recorded using a stereo microscope (Zeiss FV10) and a CCD camera (Olympus DP73) at 12.5 frames per second.

## Additional Information

**How to cite this article**: Ota, S. *et al*. Functional visualization and disruption of targeted genes using CRISPR/Cas9-mediated eGFP reporter integration in zebrafish. *Sci. Rep.* **6**, 34991; doi: 10.1038/srep34991 (2016).

## Supplementary Material

Supplementary Information

Supplementary Video

Supplementary Video

Supplementary Video
