# Supplementary material for: Functional visualization and disruption of targeted genes using CRISPR/Cas9-mediated eGFP reporter integration in zebrafish
Source: Sci Rep. 2016 Oct 11;6:34991. doi: 10.1038/srep34991 (PMC5057081; doi:10.1038/srep34991)
Supplement: Supplementary Information [file srep34991-s1.pdf]

**Supplementary Information**

# Functional visualization and disruption of targeted genes using CRISPR/Cas9-mediated eGFP reporter integration in zebrafish

Satoshi Ota[1*], Kiyohito Taimatsu[1*], Kanoko Yanagi[1], Tomohiro Namiki[1], Rie Ohga[1], Shin-ichi Higashijima[2], Atsuo Kawahara[1]

[1]Laboratory for Developmental Biology, Center for Medical Education and Sciences, Graduate School of Medical Science, University of Yamanashi, Shimokato 1110, Chuo, Yamanashi, 409-3898, Japan

[2]National Institutes of Natural Sciences, Okazaki Institute for Integrative Bioscience, National Institute for Basic Biology, Okazaki, Aichi, 444-8787, Japan

[*]These authors contributed equally to this work.

*pax2a*-target 1

mutation frequency: 6/15=40%

```
GCGAGGGGGGATCTGGGAAGGAGGGGGGCGGTGGACTGCAGCTTTT  WT x9
GCGAGGGGGGATCTGGGAAGG-GGGGGGCGGTGGACTGCAGCTTTT  -1
GCGAGGGGGGATCTGGGAAGGAG--GGGCGGTGGACTGCAGCTTTT  -2
GCGAGGGGGGATCTGGGAAGGT----GGCGGTGGACTGCAGCTTTT  -4 (-5/+1)
GCGAGGGGGGATCTGGGAAGGAG------GGTGGACTGCAGCTTTT  -6
GCGAGGGGGGATCTGGGAAGGAG---------GGACTGCAGCTTTT  -9
GCGAGGGGGGATGT-----------GGGCGGTGGACTGCAGCTTTT  -11 (-13/+2)
```

*pax2a*-target 2

mutation frequency: 1/15=6.7%

```
GGAGCAGACGCAACCACTAATGCTTGCGGTCCCTTAAA  WT x14
GGAGCAGACGCAACCACTAATG----CGGTCCCTTAAA  -4 x1
```

*pax2a*-target 3

mutation frequency: 1/13=7.7%

```
TTTCAATTATAGCTACATAATCAAGGCGAGACGGCGACCTCAGTC  WT x12
TTTCAATTATAGCTACATAATCAAGG--------CGACCTCAGTC  -8 x1
```

**Figure S1. Mutation frequencies in the injected F0 embryos.** We evaluated the genome-editing activities of *pax2a*-gRNA1-3 by examining mutation frequencies in the gRNA/Cas9-injected F0 embryos. Mutation frequencies were determined using random sequencing of PCR amplified-fragments from genomic DNAs prepared from the injected F0 embryos. The target sequences are highlighted in yellow. Blue letters indicate PAM sequences. Deleted and inserted nucleotides are indicated by red dashes and red letters, respectively. The number of nucleotides deleted and inserted is indicated to the right with the detection number.

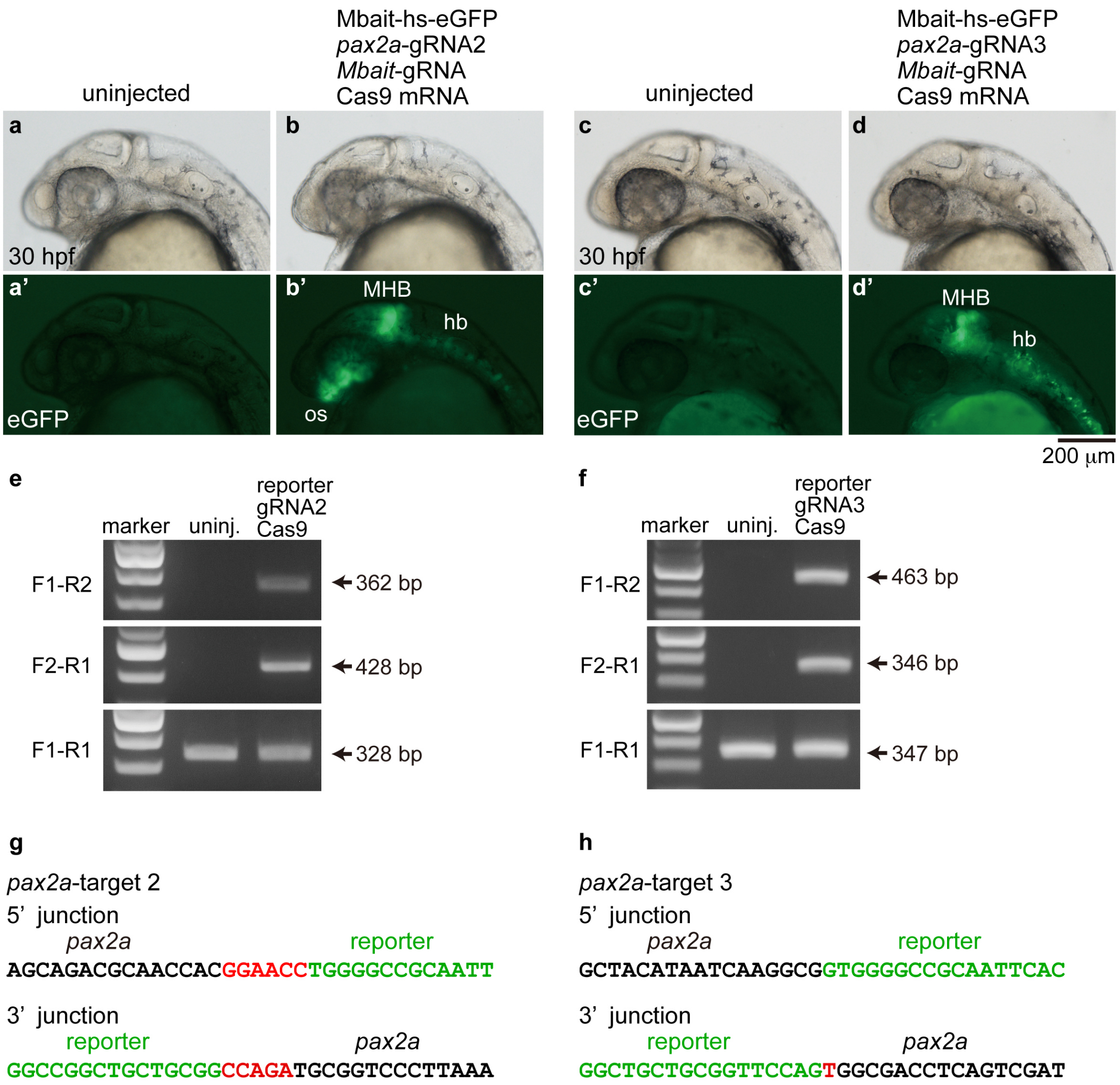


**Figure S2. Targeted genomic integration of the Mbait-hs-eGFP into the *pax2a* locus using the CRISPR/Cas9.** (a, a', c, c') Uninjected embryos. (b, b') An embryo injected with the reporter, *pax2a*-gRNA2, *Mbait*-gRNA and Cas9 mRNA. The expression of eGFP was detected in the MHB, the optic stalk (os) and the hindbrain neurons (hb). (d, d') An embryo injected with the reporter, *pax2a*-gRNA3, *Mbait*-gRNA and Cas9 mRNA. The expression of eGFP was detected in the MHB and the hindbrain neurons. (e, f) The integrations of the reporter in the *pax2a* gene (e; *pax2a*-target 2, f; *pax2a*-target 3) were determined by genomic PCR using the *pax2a*-specific and

reporter-specific primers. Targeted positions of the primers are shown in Figure 1. (g, h) Genomic sequence of the 5' junction and the 3' junction at the integration site in the eGFP-positive embryo. Inserted nucleotides are indicated in red letters. Black letters and green letters represent *pax2a* sequences and the reporter sequences, respectively.

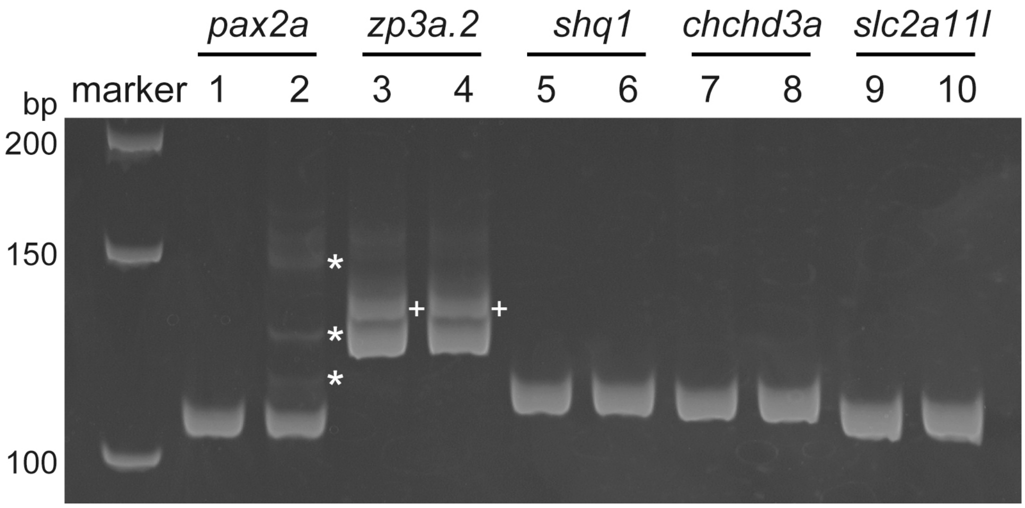


**Figure S3. Off-target effect in the *pax2a*-gRNAs/Cas9 injected embryos.** Genomic fragments containing on-target (*pax2a*) and off-target sites (*zp3a.2*, *shq1*, *chchd3a* and *slc2a11l*) were amplified from genomic DNAs (uninjected, 1, 3, 5, 7, 9; *pax2a*-gRNA1/Cas9-injected, 2, 4, 6, 8; *pax2a*-gRNA2/Cas9-injected, 10) by PCR using individual locus-specific primers (Supplementary Table S4). Heteroduplexes were detected in the PCR amplicon for *pax2a* locus, but not in the PCR amplicons for *zp3a.2*, *shq1*, *chchd3a* and *slc2a11l* loci. Asterisks (*) and crosses (+) indicate heteroduplexes and non-specific bands, respectively.

Tg[pax2a-hs:eGFP]

5’ junction

GACCTCCTCATTTGGTGCTCCAGCTTTTTGCGTTGGCGAATCACAAAGTGTTGGAATCT

target 1

ATTGCCTTTGTCTGACATGTCATCCATCTCTATGCGAGGGGGGATCTGGGAAGGCGGTG
GACTGCAGCTTTTAGAAACAGACACACTGAGAAGAGGGGTTTCAGTCTCAGCCCAAAGC
TTCTAACAGGCACATCCCATTCTTGAAGAGAGGGGGACTGAGAGACACTCGCCGTGTCC
ATGTTTTTTCCTGTCATTCCCGCACGAATCTGACTGTACCCCTCAGACTGTTCAGACCA
GAAACGCTTCTCCACATCGAGGAATTTACACTGCAGTTTCACCAGAGGACACTGGAGCA

target 2 Mbait donor vector

GACGCAACCACTAATGCGGTGGGGCCGCAATTCACTGGAGGCTTCCAGAACAGTGTAGA
GTCTGAACGGGTGCGCGCTCTGCTGTATTTAAAGGGCGAAAGAGAGACCGCAGAGAAAC

3’ junction

donor vector

TTATGCTTCCGGCTCGTATGTTGTGTGGAATTGTGAGCGGATAACAATTTCACACAGGA
AACAGCTATGACCATGATTACGCCAAGCGCGCAATTAACCCTCACTAAAGGGAACAAAA

Mbait target 2

GCTGGAGCTCCACCGCGGTGGCGGCCGGCTGCTGCCGGCTGCTTGCGGTCCCTTAAATA
TGTAGCTAACAAATTTTTACTCAACGACTGATTACGCTAACGTTCCTGCGTGAAAGATT
TTTGAGAGACCAGTGCAGGACATGATCTGCACCTGACCAGCCTCGTGTCTTCGTTGAAT
GGACTGATCAGCACGAGATAAAGTTCGTCGCGCGTTGAACAACTTCTTCTGTACACTGG

target 3

CATTGGAAGCACCGGGCTTTGAACGGATAGCGATCTTTATTAATTTCAATTATAGCTAC
ATAATCAAGGCGAGACGGCGACCTCAGTCGATTATCTTTCCTTCCTTTGCTATCCCATG
GATATTC

target 1

| | |
|---|---|
| WT | GGGGATCTGGGAAGGAGGGGGGCGGTGGACTG |
| Tg[pax2a-hs:eGFP] | GGGGATCTGGGAAGG-------CGGTGGACTG -7 |

target 3

| | |
|---|---|
| WT | TAGCTACATAATCAAGGCGAGACGGCGACCT |
| Tg[pax2a-hs:eGFP] | TAGCTACATAATCAAGGCGAGACGGCGACCT |

**Figure S4. Sequence of the knock-in allele in the Tg[pax2a-hs:eGFP] embryo.** A sequence analysis revealed that Mbait-hs-eGFP was integrated into the *pax2a*-target 2. The *pax2a*-target 1 had a 7-bp deletion, and the *pax2a*-target 3 had no indel mutation. The *pax2a*-target sites are highlighted in yellow. The Mbait sequences are highlighted

in magenta. Blue letters indicate PAM sequences. A red dash indicates a nucleotide deleted in the target locus. Green letters represent the donor vector sequences.

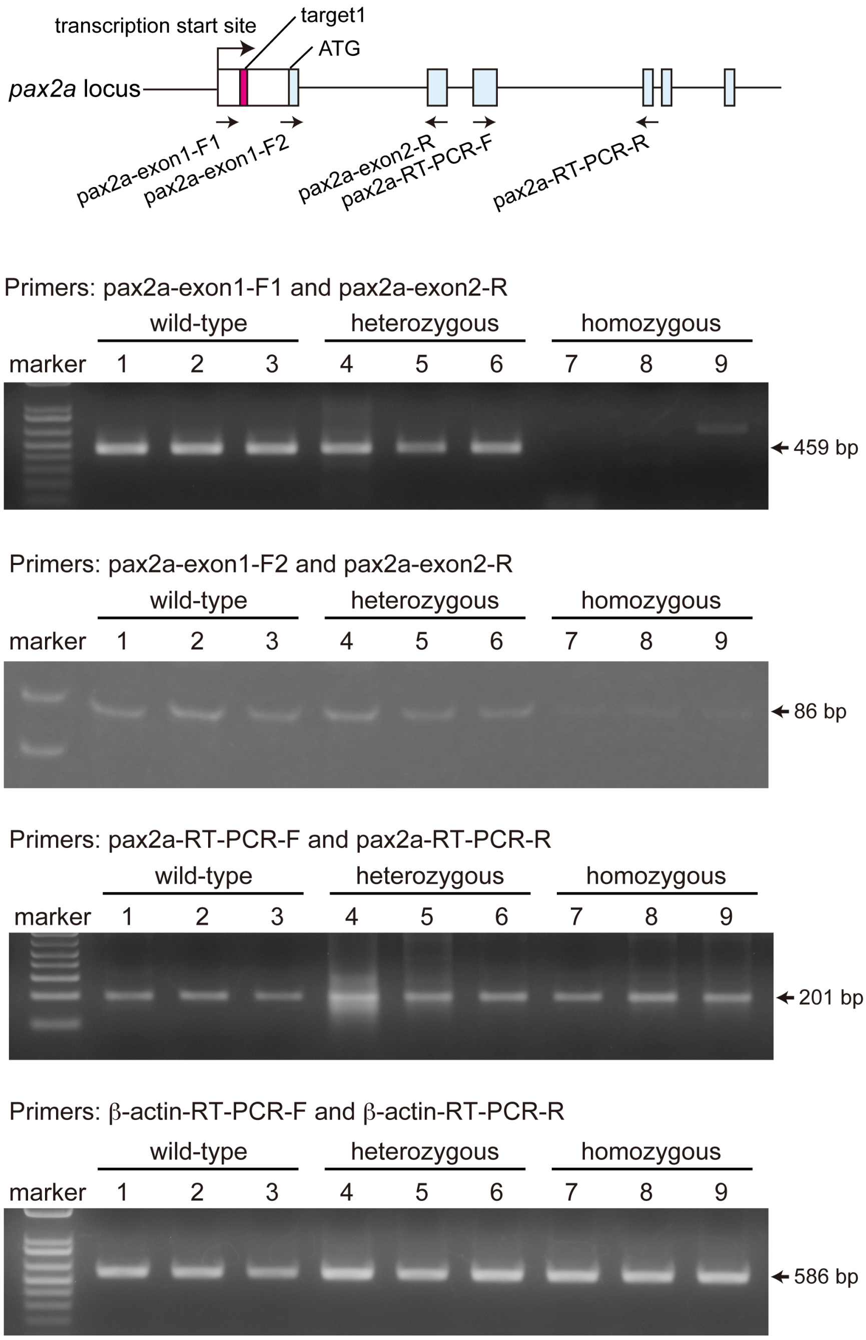


**Figure S5. RT-PCR to detect the *pax2a* transcripts.** A schematic representation of the *pax2a* locus and primers positions (Top). Total RNAs were prepared from wild-type

(1-3), heterozygous Tg[pax2a-hs:eGFP] (4-5) and homozygous Tg[pax2a-hs:eGFP] (7-9) embryos. The *pax2a* transcripts were amplified by RT-PCR using the locus-specific primers (Supplementary Table S4). The *pax2a* transcripts containing exon 1 and exon 2 were suppressed in the homozygous embryos, but not wild-type and the heterozygous embryos. The *pax2a* transcripts containing exon 3 and exon 4 were comparably detected in all embryos. The *β-actin* transcripts were amplified by RT-PCR using the *β*-actin-RT-PCR-F and the *β*-actin-RT-PCR-R primers (Supplementary Table S4). PCR amplicons (86 bp) using pax2a-exon1-F2 and pax2a-exon2-R were separated by polyacrylamide gels, other PCR amplicons were separated by agarose gels.

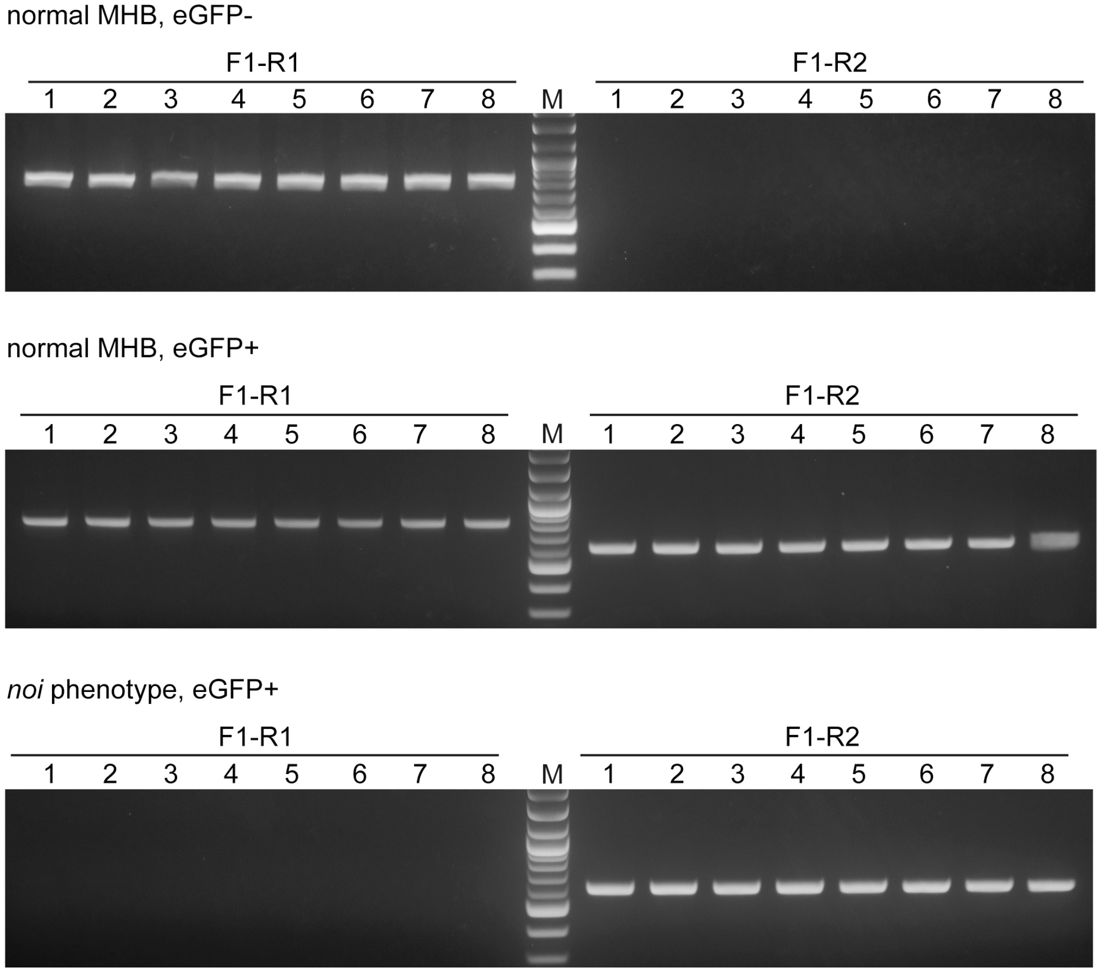

**Figure S6. Genotyping of Tg[pax2a-hs:eGFP] F2 embryos obtained from mating heterozygous Tg[pax2a-hs:eGFP] F1 fish.** We classified F2 embryos into three groups, groups; group 1, eGFP negative (eGFP-) and normal MHB; group 2, eGFP positive (eGFP+) and normal MHB; group 3, eGFP+ and *no isthmus* (*noi*) phenotype. Eight embryos in each group were genotyped. As the result of PCR-based genotyping using the *pax2a*-specific and reporter-specific primers (Fig. 1a), we verified that group 1 was wild-type, group 2 was heterozygous Tg[pax2a-hs:eGFP], and group 3 was homozygous Tg[pax2a-hs:eGFP]. M; molecular weight marker.

*epdr1*-target

mutation frequency: 16/19=84.2%

| | |
|---|---|
| CGTCATTATTGCGGTGAACTAGCAGAGATGTTGGTGT | WT x3 |
| CGTCATTATTGCGGTGAACTAGCAGAGTTGTTGGTGT | 0 (-1/+1) x2 |
| CGTCATTATTGCGGTGAACTAGCAGAG---TTGGTGT | -3 x1 |
| CGTCATTATTGCGGTGAACTAGCA---ATGTTGGTGT | -3 x1 |
| CGTCATTATTGCGGTGAACTAGC-----TGTTGGTGT | -5 x1 |
| CGTCATTATTGCGGTGAACTAAC-----TGTTGGTGT | -5 (-7/+2) x9 |
| CGTCATTATTGCGGTGAACTAGCAG-----TTGGTGT | -5 x1 |
| CGTCATTATTGCGGTGAACTAGCAGAGAGTGTTGGTGT | +1 x1 |

**Figure S7. Mutation frequencies in the injected F0 embryos.** We evaluated the genome-editing activities of *epdr1*-crRNA by examining mutation frequencies in the injected F0 embryos. Mutation frequencies were determined using random sequencing of PCR amplified-fragments from genomic DNAs prepared from the injected F0 embryos. The target sequences are highlighted in yellow. Blue letters indicate PAM sequences. Deleted and inserted nucleotides are indicated by red dashes and red letters, respectively. The number of nucleotides deleted and inserted is indicated to the right with the detection number.

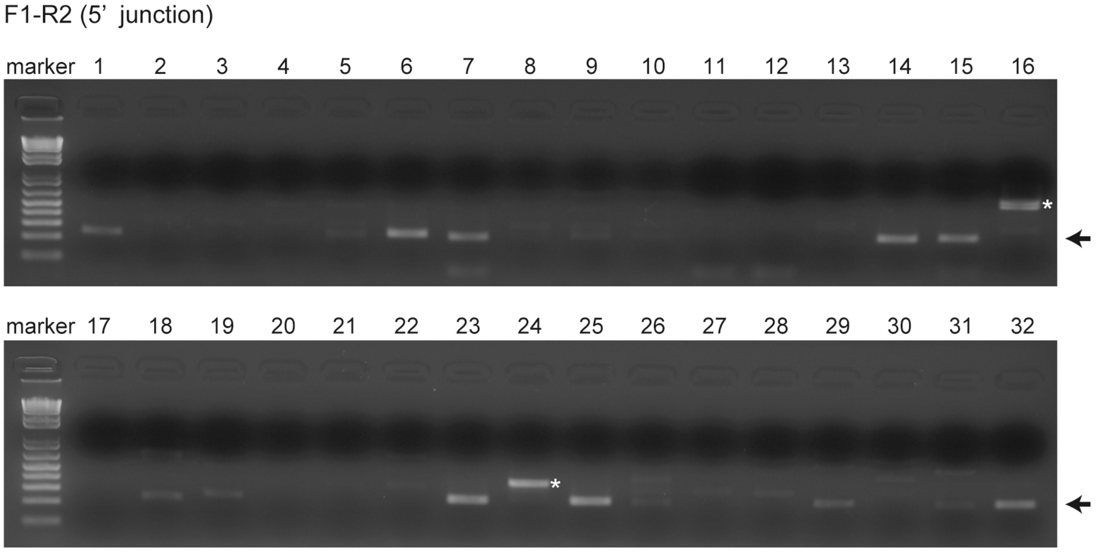


**Figure S8. Genomic PCR to assess the integration events into the *epdr1* locus.** The genomic DNAs were prepared from 32 embryos injected with *epdr1*-crRNA, tracrRNA, Cas9 protein and the donor vector. PCR amplicons using the epdr1-F1 and the reporter-R2 primers (Supplementary Table S4), were electrophoresed in 2% agarose gels. The expected bands (around 200 bp) and larger bands (16, 24) are indicated by arrows and asterisks, respectively. Sequence analysis revealed that the expected bands (arrows) contained the donor vector sequences, while larger bands (asterisks) did not contain the *epdr1* genomic sequences. The genomic integration frequencies of the reporter into the *epdr1* locus was 21.9% (7 positive-embryos in 32 embryos)

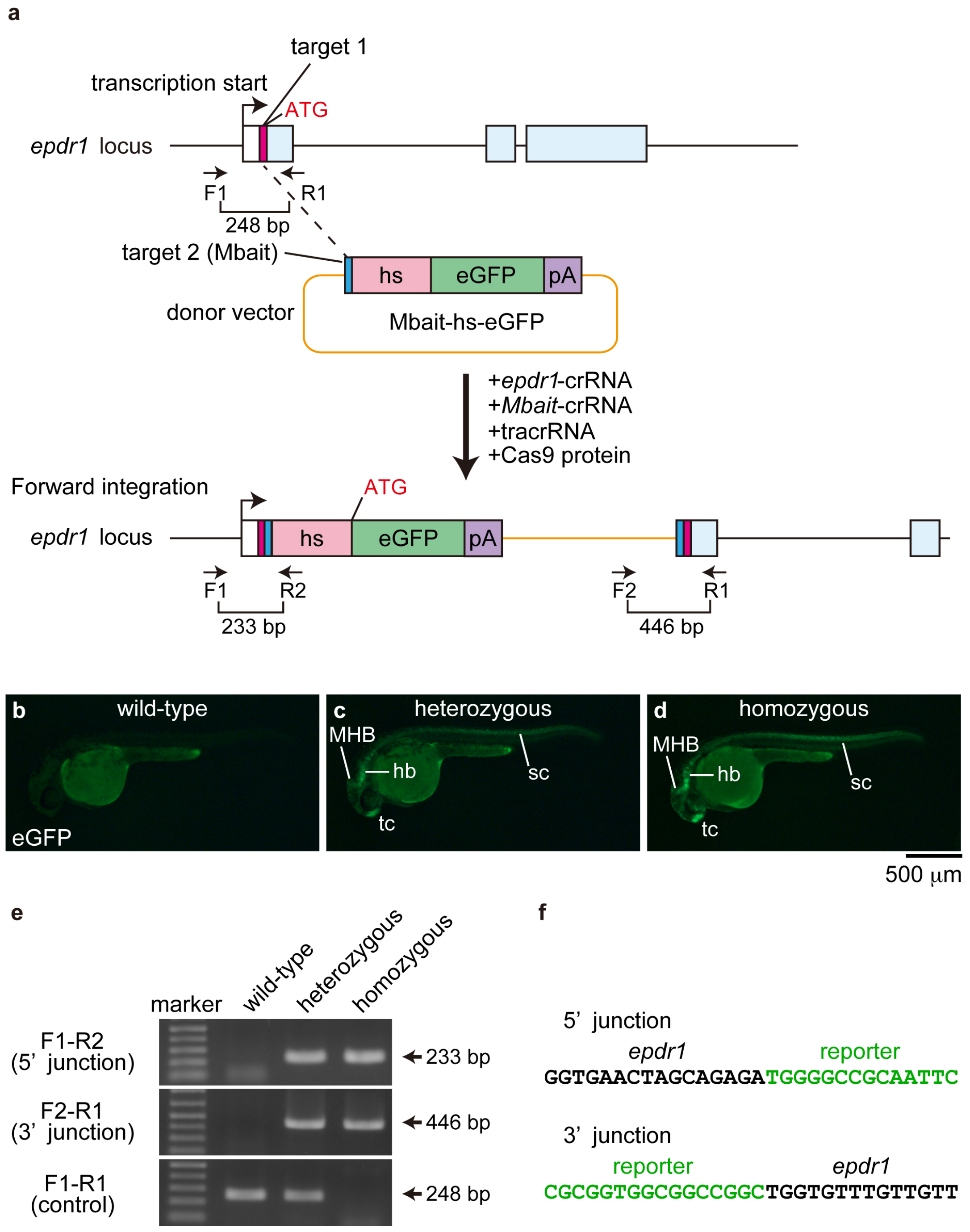


**Figure S9. The eGFP expression in Tg[epdr1-hs:eGFP] embryos.** (a) A schematic representation of the *epdr1* locus containing the 5' untranslated region (white box) and the *epdr1*-crRNA target site (pink box), and the reporter construct consisting of the *Mbait*-crRNA target site (blue box), the hsp70 promoter (hs, light red box), the eGFP gene (green box) and the polyA signal (pA; purple box). (b-d) eGFP expression in

wild-type, heterozygous and homozygous Tg[epdr1-hs:eGFP] embryos. (e) The integration of the reporter into the *epdr1* locus was determined by genomic PCR using the *epdr1*-specific and the reporter-specific primers. (f) Sequence of the 5' junction and the 3' junction at the integration site of Tg[epdr1-hs:eGFP]. Black letters and green letters represent *epdr1* sequences and reporter sequences, respectively.

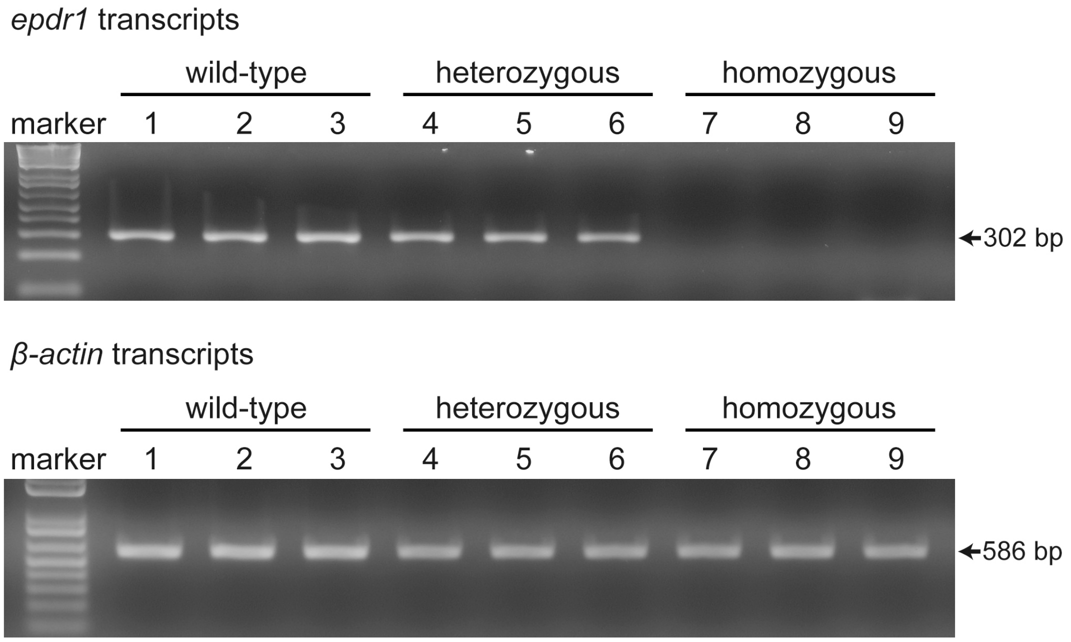


**Figure S10. RT-PCR to detect the *epdr1* transcripts containing exon 1 and exon 2.** Total RNAs were prepared from wild-type (1-3), heterozygous Tg[epdr1-hs:eGFP] (4-5) and Tg[epdr1-hs:eGFP] homozygous (7-9) embryos. The *epdr1* transcripts containing exon 1 and exon 2 were amplified by RT-PCR using the epdr1-exon1-F and the epdr1-exon2-R primers (Supplementary Table S4). The *β-actin* transcripts were amplified by RT-PCR using the *β*-actin-RT-PCR-F and the *β*-actin-RT-PCR-R primers (Supplementary Table S4).

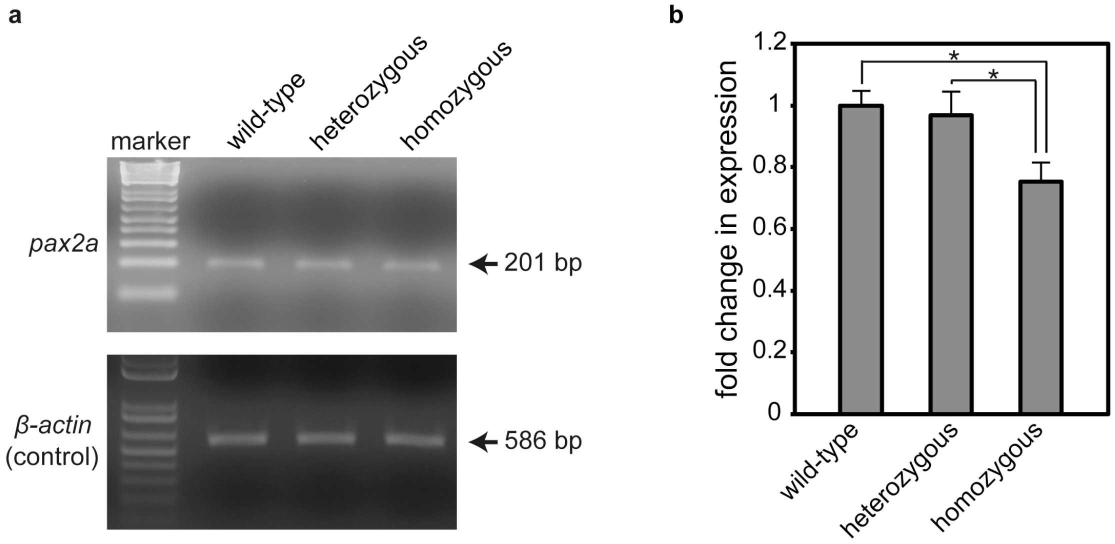


**Figure S11. Semi-quantitative RT-PCR of *pax2a* transcripts.** (a) Semi-quantitative RT-PCR to assess the *pax2a* transcripts and *β-actin* transcripts. Total RNAs were prepared from wild-type, heterozygous Tg[epdr1-hs:eGFP] and homozygous Tg[epdr1-hs:eGFP] embryos. The *pax2a* transcripts were amplified by RT-PCR using the pax2a-RT-PCR-F and the pax2a-RT-PCR-R primers (Supplementary Table S4). The *β-actin* transcripts were amplified by PCR using the *β*-actin-RT-PCR-F and the *β*-actin-RT-PCR-R primers (Supplementary Table S4). (b) Fold change in *pax2a* expression relative to internal standard (*β-actin*). We performed four times the RT-PCR experiments with triplicate samples. The bar graph shows the ratio of *pax2a* to *β-actin* transcripts. Error bars indicate standard error of the mean (SEM). Statistical significance was determined using Student's t-test. *P<0.05.

**Supplementary Movie 1** wild-type at 2 dpf.

**Supplementary Movie 2** heterozygous Tg[epdr1-hs:eGFP] at 2 dpf.

**Supplementary Movie 3** homozygous Tg[epdr1-hs:eGFP] at 2 dpf

Table S1. The genomic integration frequencies of the reporter into the *pax2a* locus.

| Targeting system | Number of eGFP expressing embryos/Number of the injected embryos | Number of eGFP expressing embryos in the *pax2a* expression domains/Number of the injected embryos |
|---|---|---|
| *pax2a*-gRNA1 Cas9 | 145/330 (44%) | 4/330 (1.2%) |
| *pax2a*-gRNA2 Cas9 | 67/179 (37%) | 4/179 (2.2%) |
| *pax2a*-gRNA3 Cas9 | 42/143 (29%) | 2/143 (1.4%) |
| *pax2a*-gRNA1/2/3 Cas9 | 34/42 (81%) | 2/42 (5%) |

Table S2. Germline transmission of Tg[pax2a-hs:eGFP] and Tg[epdr1-hs:eGFP].

| Knock-in line | Number of fish screened | Number of fish integrated | Germline transmission frequency in F1 embryos |
|---|---|---|---|
| Tg[pax2a-hs:eGFP] | 20 | 1 | 46/142 (32%) |
| Tg[epdr1-hs:eGFP] | 58 | 1 | 102/224 (46%) |

Table S3. The targeted genomic sequences and oligonucleotides for the construction of gRNA expression vectors.

Targeted genomic sequences for CRISPR/Cas9

| Target | Sequences (5’ to 3’) |
|---|---|
| *pax2a*-1 | GGATCTGGGAAGGAGGGGGGCGG |
| *pax2a*-2 | GACGCAACCACTAATGCTTGCGG |
| *pax2a*-3 | GCTACATAATCAAGGCGAGACGG |
| *epdr1* | CGGTGAACTAGCAGAGATGTTGG |

PAM sequences are underlined.

Oligonucleotides for the construction of gRNA expression vectors

| Name | Sequence (5’ to 3’) |
|---|---|
| pax2a-gRNA1-s | TAGGATCTGGGAAGGAGGGGGG |
| pax2a-gRNA1-as | AAACCCCCCCTCCTTCCCAGAT |
| pax2a-gRNA2-s | TAGGCGCAACCACTAATGCTTG |
| pax2a-gRNA2-as | AAACCAAGCATTAGTGGTTGCG |
| pax2a-gRNA3-s | TAGGTACATAATCAAGGCGAGA |
| pax2a-gRNA3-as | AAACTCTCGCCTTGATTATGTA |

Table S4. PCR primers used in this study.

| Primer name | Sequence (5' to 3') |
|---|---|
| pax2a-target 1-F1 | CATTTCCCTGTTTGCATCCCTCCC |
| pax2a-target 1-R1 | GTCAGATTCGTGCGGGAATGACAG |
| pax2a-target 2-F1 | TTCTTGAAGAGAGGGGGACTGAGAG |
| pax2a-target 2-R1 | TCTCGTGCTGATCAGTCCATTCAAC |
| pax2a-target 3-F1 | CCACTAATGCTTGCGGTCCCTTAAA |
| pax2a-target 3-R1 | AGGGGTCTGCTTTGCAGTGAATATC |
| epdr1-F1 | GCCTCTGAATCGTTGCGTGA |
| epdr1-R1 | GACCCTGATTCTCTGGTTCTGG |
| reporter-F2 | GTTGGCCGATTCATTAATGCAGCTG |
| reporter-R2 | ACACCTCGTCGGGGAAAAAGTCCTG |
| M13-F | GTAAAACGACGGCCAGT |
| M13-R | CAGGAAACAGCTATGAC |
| atp1a3a-F | CGGGATCCATGGGGTATGGACGGTCGG |
| atp1a3a-R | GCTCTAGAGTAGCCCAGGATGATGGACAG |
| zp3a.2-HMA-F | TGGTGTCCTGCTGTAGTTTCT |
| zp3a.2-HMA-R | GGCAACACCGCTGGTTTATA |
| shq1-HMA-F | AGCTTCTTGTGTGGAGCTCC |
| shq1-HMA-R | AGCAGAGCGTCTTTCCTGAG |
| chchd3a-HMA-F | GTTGATCCTCTGCCAGCACT |
| chchd3a-HMA-R | GTGGTCACTTGCTCAGGGAG |
| slc2a11l-HMA-F | TGGAGTGACAGCACCACTTC |
| slc2a11l-HMA-R | CATGCCCACTAGGAAGAGTCC |
| pax2a-RT-PCR-F | CAGAATACAAGCGGCAAAATCCT |
| pax2a-RT-PCR-R | CTATAGTGTGTCCCGCTGTGG |
| β-actin-RT-PCR-F | CTGGCCTCCCTGTCCACCTT |
| β-actin-RT-PCR-R | ACAGGTTGGCCCCACCAAAT |
| pax2a-exon1-F1 | CCACATCGAGGAATTTACACTGC |
| pax2a-exon1-F2 | ATGGATATTCACTGCAAAGCAGAC |
| pax2a-exon2-R | CCATTCACAAACACCCCTCCTAG |
| epdr1-exon1-F | CGTCATTATTGCGGTGAACTAGC |
| epdr1-exon2-R | TCCTCAATCTGAAATAGCACTCCA |
